# Curcumin Retains Anti-Inflammatory Effects Despite IDO Inhibition-Associated Neutrophilic Shift in OVA-Induced Rat Model

**DOI:** 10.3390/biom16091295

**Published:** 2026-09-08

**Authors:** Mubeen Fatima, Ali Rafi, Muhammad Shoaib Zafar, Usman Aftab, Khush Bakhat Kiran, Muhammad Shahzad, Safdar Hussain, Hongbo Wang

**Affiliations:** 1 Centre for Applied Molecular Biology, Sector 3, West 87 Canal Rd, Quaid-i-Azam Campus, University of the Punjab, Lahore 53700, Pakistan; mubiifatima@gmail.com; 2 Department of Forensic Sciences, Lahore University of Biological and Applied Sciences, Lahore 53400, Pakistan; 3 Department of Pharmacology, University of Health Sciences, Lahore 54600, Pakistan or shahzad912@hotmail.com (M.S.); 4 College of Pharmacy, University of Health Sciences, Lahore 54600, Pakistan; shoaibzafar786@gmail.com; 5 Department of Human Anatomy, School of Basic Medicine, Shenyang Medical College, No. 146 Huanghe North Street, Shenyang 110034, China; 6 Liaoning Province Key Laboratory for Phenomics of Human Ethnic Specificity and Critical Illness, Shenyang 110034, China

**Keywords:** curcumin, Indoleamine 2,3-dioxygenase, 1-methyl-DL-tryptophan, airway inflammation, immunomodulation, neutrophils

## Abstract

**Highlights:**

- Does curcumin (CN) maintain its anti-inflammatory efficacy when IDO is pharmacologically blocked?
  - CN retains its anti-inflammatory effects even when IDO is pharmacologically inhibited.
- Does CN suppress both inflammatory cell infiltration and inflammatory mediator expression?
  - CN effectively suppresses both inflammatory cell infiltration and the expression of inflammatory mediators.
- Does CN reduce the expression of key inflammatory genes?
  - CN decreases the expression of major inflammatory genes, including *TNF-α*, *IL-4*, *IL-6*, and *Cxcr2*. The expression of *Nfkb1* was not consistently retained under pharmacological IDO inhibition.
- Does CN protect lung structure against inflammatory injury when IDO is pharmacologically blocked?
  - CN reduces histopathological lung injury and pulmonary edema, thereby providing protection against inflammation associated structural damage.

**Abstract:**

Allergic asthma is characterized by Th2-driven airway inflammation. Indoleamine 2,3-dioxygenase (IDO) maintains immune tolerance, but its role in modulating the anti-inflammatory actions of Curcumin (CN) is unclear. This study investigated whether CN retains efficacy in a rat model of Ovalbumin (OVA)-induced airway inflammation under pharmacological IDO inhibition. Rats were sensitized and challenged with OVA and treated with methylprednisolone (MP, 15 mg/kg), CN (200 mg/kg), the IDO inhibitor 1-MT (70 mg/kg), or CN+1-MT. Assessments included systemic and pulmonary leukocyte profile, delayed-type hypersensitivity (DTH), OVA-specific IgE, bronchoalveolar lavage fluid (BALF) inflammatory cells and nitric oxide (NO) levels, lung wet/dry weight ratio, gene expression of tumor necrosis factor alpha (TNF-α), interleukin (IL)-4, IL-6, *Cxcr2* and transcription factor Nfkb1 and lung histopathology. CN alone or combined with 1-MT normalized leukocyte counts, suppressed eosinophilic and neutrophilic infiltration, restored BALF NO levels, reduced DTH response and OVA-specific IgE, decreased pulmonary edema, and preserved pulmonary vascular integrity. CN significantly attenuated TNF-α, IL-4, IL-6, and *Cxcr2* gene expression despite pharmacological IDO inhibition. Histopathology revealed reduced inflammatory infiltration and maintained alveolar structure. CN maintains anti-inflammatory, immunomodulatory and tissue-protective efficacy in allergic airway inflammation despite pharmacological IDO inhibition. These findings call for future research on CN as a potential adjunctive therapy method for inflammatory airway diseases. However, to elucidate the underlying processes, more research on IDO activity and downstream immunometabolic pathways is needed.

## 1. Introduction

Asthma and chronic obstructive pulmonary disease (COPD) are characterized by airway inflammation, tissue remodeling and airway obstruction, contributing to global morbidity and healthcare burden [1]. Allergic airway inflammation is a multifaceted process involving the infiltration of neutrophils, eosinophils, and T lymphocytes into airway mucosa, driving a cascade of pathological events that culminate in airway obstruction, mucus hypersecretion and tissue remodeling [2].

Allergic asthma pathogenesis is predominantly characterized by the dysregulation of adaptive immunity, particularly the dominance of T-helper 2 (Th2)-mediated responses. Th2-dominant immunity orchestrates the allergic cascade through cytokines like interleukin-4 (IL-4), which is pivotal for IgE class switching in B cells, Th2 cell differentiation, and eosinophil recruitment [3]. This cytokine milieu is further intensified by the activity of additional pro-inflammatory mediators, including IL-6, *Cxcr2*, and TNF-α, along with transcriptional regulator nuclear factor kappa B (NF-κB), which collectively amplify and sustain airway inflammation [4]. Neutrophil-predominant inflammation is increasingly recognized in severe, steroid-resistant asthma and COPD, associated with tissue damage from reactive oxygen species, proteolytic enzymes, and profibrotic mediators [5].

Indoleamine 2,3-dioxygenase (IDO) is essential for establishing immune tolerance in mucosal tissues. It achieves this by converting tryptophan in to kynurenine, which inhibits the overactivation of T cells and fosters the differentiation of regulatory T-cell cells [6,7]. IDO deficiency or pharmacological inhibition enhances Th1/Th17 responses, promotes neutrophil recruitment, and impairs the resolution of inflammation [7,8].

The cornerstone of pharmacological management for persistent asthma and airway diseases is inhaled corticosteroids (ICS), often combined with long-acting β2-agonist (LABA), which activate glucocorticoid receptors in lung tissues to exert anti-inflammatory effects [9,10]. Despite their central role, disease control remains suboptimal in many patients due to systemic adverse effects, diminished responsiveness, and poor treatment adherence [11,12]. This therapeutic gap has driven interest in complementary and alternative medicines, with herbal remedies gaining popularity for their historical use and perceived safety [13].

Curcumin (CN), a bioactive polyphenol from *Curcuma longa*, has potent anti-inflammatory and immunomodulatory activities [14,15]. CN is a lipophilic diarylheptanoid with limited oral bioavailability due to fast glucuronidation/sulfation, significant first-pass metabolism, and low water solubility [16]. Preclinical and clinical studies show a favorable safety profile at regularly examined dosages, with the most often reported side effects being mild gastrointestinal disruption (nausea and diarrhea) at large oral doses; significant toxicity is uncommon [17]. Mechanistically, CN inhibits NF-κB signaling cascade by preventing the IκBα phosphorylation and subsequent p65 nuclear translocation, while concurrently suppressing mitogen-activated protein kinase (MAPK) pathways [18]. These actions downregulate the transcription of pro-inflammatory cytokines (e.g., TNF-α, IL-1β, IL-6), chemokines and matrix metalloproteinases, mitigating inflammatory cell recruitment, mucus hypersecretion, and airway damage [19,20]. Despite extensive evidence of CN’s anti-inflammatory properties, its efficacy under impaired IDO-mediated tolerance remains unclear.

While CN’s anti-inflammatory efficacy in Th2-driven airway inflammation is well documented, whether this efficacy is dependent on intact IDO-mediated immunoregulation relevant to steroid-resistant or neutrophil-predominant asthma phenotypes in which IDO activity is reduced has not been previously tested and is the specific gap addressed here. The present study investigated whether CN retains its anti-inflammatory and tissue-protective effects in an OVA-induced allergic airway inflammation model when IDO is pharmacologically inhibited with 1-MT. Rather than exploring the molecular basis of IDO signaling, this study aimed to find out if CN remained therapeutic when IDO activity is pharmacologically inhibited.

## 2. Materials and Methods

### 2.1. Study Setting and Animals

This study was a collaborative effort, with experiments carried out jointly at the facilities of the Center for Applied Molecular Biology (University of the Punjab, Lahore, Pakistan) and the Department of Pharmacology (University of Health Sciences, Lahore, Pakistan). Male Wistar rats aged 6–8 weeks, weighing 160–180 g, were sourced from the Experimental Research Lab at UHS Lahore. Following acquisition, the animals underwent a 7-day acclimatization period under the controlled environmental conditions, maintaining a temperature of 25 ± 2 °C, 45–65% humidity, with a balanced 12 h cycle of light and darkness. The rats received standard laboratory rodent diet and drinking water *ad libitum*. All study protocols were formally approved by the Institutional Ethical Review Board of the University of the Punjab (Approval No. D/70/FIMS, dated 15 May 2023), with all procedures strictly conforming to national and international standards for laboratory animal welfare.

### 2.2. Chemicals and Reagents

Ovalbumin (OVA; Cat. #A5503), CN (CN; Cat. #C1386), and 1-methyl-DL-tryptophan (1-MT; Cat. #447439) were purchased from Sigma-Aldrich (St. Louis, MO, USA). Methylprednisolone (MP) was purchased from Pfizer Pakistan Ltd. (Karachi, Pakistan). Nitric oxide (NO) levels were measured using the Griess Reagent Kit (Biotium, Fremont, CA, USA). Serum OVA-specific IgE concentrations were determined using the Rat Ovalbumin-Specific IgE ELISA Kit (MyBioSource, San Diego, CA, USA; Cat. #MBS702479). Total RNA was extracted using TRIzol^®^ Reagent (Invitrogen^TM^, Thermo Fisher Scientific, Waltham, MA, USA; Cat. # 15596026), and complementary DNA (cDNA) was generated using the RevertAid First Strand cDNA Synthesis Kit (Thermo Fisher Scientific, Waltham, MA, USA; Cat. # K1622). Quantitative real-time PCR was conducted using 2 X SYBR Green PCR Master Mix (Thermo Fisher Scientific, Waltham, MA, USA; Cat. # F- 416L).

### 2.3. Experimental Design and Groups

This study comprised a total of 48 adult male Wistar rats. The sample size of eight animals per group (*n* = 8) was specified in the study design at the time of ethical approval, based on the six-group experimental design and group sizes utilized in comparable OVA-induced airway inflammation experiments in Wistar rats. An a priori power analysis of BALF lymphocyte data from Zafar et al. (2022) indicated that *n* = 8 per group was sufficient to obtain the primary result [21]. Thus, the power analysis confirmed the pre-specified sample size as compared to determining it retrospectively. The sample size was chosen to maintain statistical adequacy, predicted biological variation, and the reduction concept of the 3Rs paradigm [21,22]. Following the acclimation phase, the 48 rats were randomly assigned to six experimental groups (*n* = 8 each) using the lottery (chit-drawing) approach. Each rat was allocated a unique identification number, and numbered chits were chosen at random to assign animals to the appropriate experimental groups, reducing allocation bias. The control group (CG) received sham sensitization and intranasal challenge with phosphate-buffered saline (PBS). Allergic airway inflammation was induced in Ovalbumin (OVA) group and remaining treatment groups by intraperitoneal sensitization and intranasal OVA challenge. The neutrophil-predominant phenotype in the present research results from pharmacological IDO inhibition superimposed on the OVA model and should be interpreted as a substitute characterization rather than a validated neutrophilic-asthma model because no specific neutrophilic-asthma induction protocol (such as LPS or house dust mites) was used. Four treatment groups were included: MP (standard of care comparator, 15 mg/kg, i.p.), CN (200 mg/kg, i.p.), 1-MT (IDO inhibitor, 70 mg/kg, i.p.) CN+1-MT (combined CN 200 mg/kg and 1-MT 70 mg/kg, i.p.). Ovalbumin (OVA) was dissolved in sterile phosphate-buffered saline (PBS) just before sensitization and intranasal challenge. CN (200 mg/kg body weight) was prepared just before being administered by dissolving it in 5% (*v*/*v*) dimethyl sulfoxide (DMSO) and diluting with sterile distilled water to the optimal concentration. 1-Methyl-DL-tryptophan (70 mg/kg body weight) and methylprednisolone (15 mg/kg body weight) were diluted in sterile distilled water just before usage. All therapies were delivered intraperitoneally (i.p.) following previously confirmed methods [21,22,23].

### 2.4. Sensitization and Challenge Protocol

**Figure 1:**
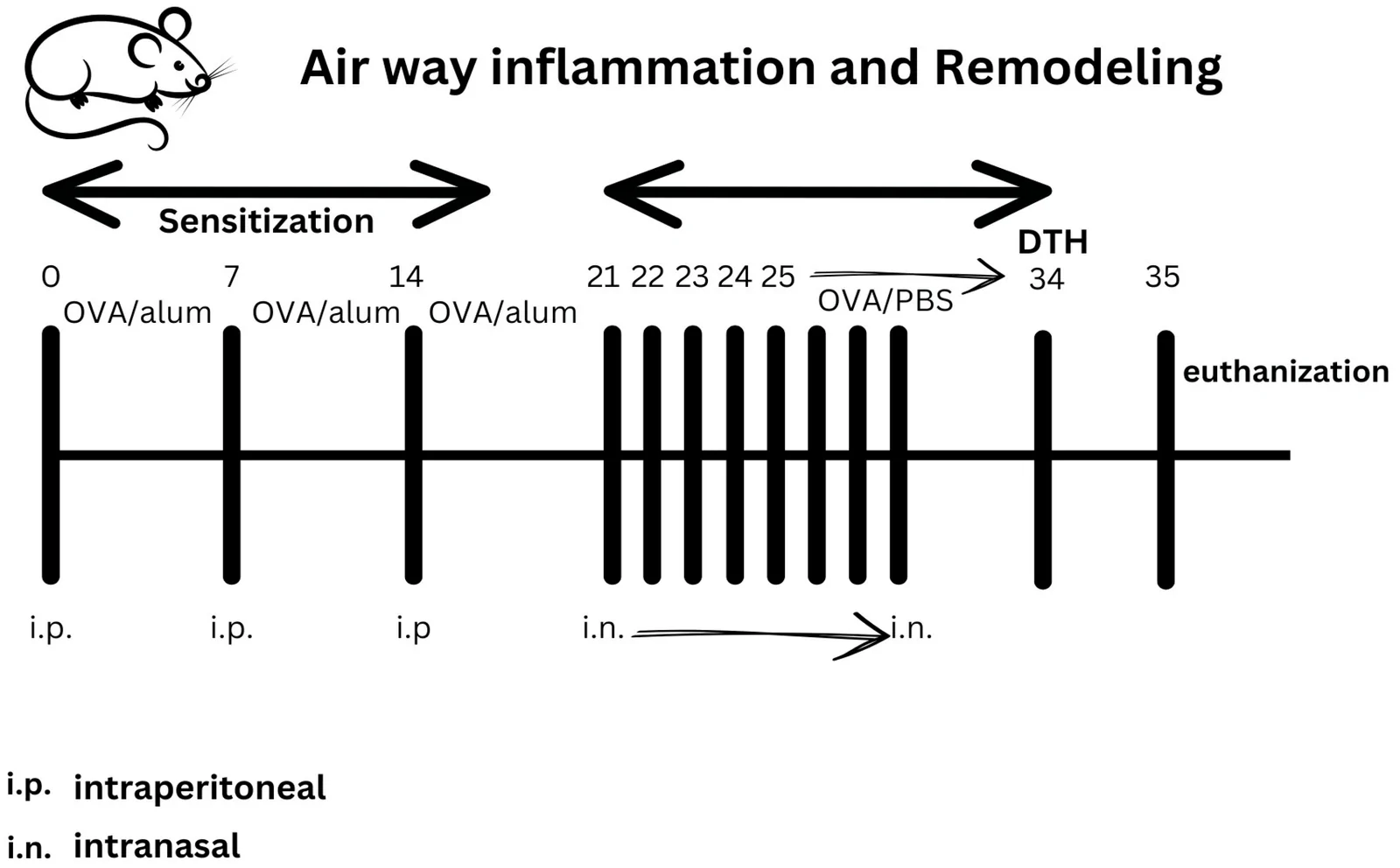
Experimental timeline of OVA-induced airway inflammation and therapeutic intervention. Numbers represent experimental days. Rats were sensitized intraperitoneally with Ovalbumin (OVA) + aluminum hydroxide adjuvant (alum) on days 0, 7, and 14, followed by daily intranasal OVA challenge from days 21 to 34, with delayed-type hypersensitivity (DTH) measured on day 34; tissue and fluid were collected on day 35. Therapeutic drugs (methylprednisolone, CN, 1-methyl-DL-tryptophan, or CN+1-MT) were given intraperitoneally 2 h before to each intranasal challenge.

Airway inflammation was induced as previously described [22,24], with a schematic timeline shown in Figure 1. Briefly, rats were sensitized via i.p. injection of 200 µL emulsion containing 1 mg OVA and 1 mg aluminum hydroxide (alum). The CG received PBS with alum instead of OVA. Beginning on day 21 and continuing through day 34, animals received daily intranasal administration of OVA solution, prepared by dissolving 1 mg of OVA in 1 mL of PBS, while CG received intranasal PBS alone. Therapeutic agents were administered i.p. daily, 2 h before intranasal OVA challenge (Figure 1).

### 2.5. Delayed-Type Hypersensitivity (DTH) Assay

Twenty-four hours before euthanasia, the in vivo T-cell-mediated inflammatory responses were measured via a delayed-type hypersensitivity (DTH) ear assay [25]. A volume of 200 μL OVA solution (1 mg/mL) in PBS was prepared, and 20 μL was administered by slow intradermal injection into the right pinna of each rat by using insulin syringe, while the left ear received 20 μL PBS as vehicle control in the same way. After 24 h at euthanasia, both ears were excised at an identical anatomical site using a 6 mm biopsy punch and weighed once. An assessor who was not aware of the group assignment performed ear excision and weighing. The magnitude of the DTH response was expressed as the difference in mass (g) between the OVA and PBS-injected ears.

### 2.6. Hematological Analysis and Bronchoalveolar Lavage Fluid (BALF) Collection

On day 35, 24 h after the last challenge, rats were anesthetized with mild ether vapor per the approved institutional protocol (Approval No. D/70/FIMS), and blood was collected via intracardiac puncture for hematological analysis. The animals were subsequently euthanized by exsanguination during deep anesthesia, followed by thoracotomy. The trachea was then cannulated, and the lungs were lavaged three times with 2 mL of ice-cold PBS. The recovered BALF was centrifuged (1500× *g*, 10 min, 4 °C), and the resulting pellet was resuspended in 1 mL of ice-cold PBS. Total and differential leukocyte counts in blood and BALF samples were then determined using an automated hematology analyzer Sysmex XT-1800i, (Sysmex Corporation, Kobe, Japan) [26].

### 2.7. Determination of BALF Nitric Oxide (NO) Levels

NO levels in BALF were assessed by measuring the nitrite levels through the Griess reagent method employing a commercial kit (Biotium, USA). Briefly, 100 µL of BALF was incubated in a 96-well plate with 20 µL of 0.31 M phosphate buffer (pH 7.5), 10 µL NADPH (0.86 mM), 10 µL FAD (0.11 Mm) and 10 µL nitrate reductase (1 U/mL) for 1 h at room temperature to convert nitrate to nitrite. Subsequently, 20 µL Griess reagent and 130 µL deionized water were added, and the plate was incubated for 30 min. Sulfanilic acid reacts with nitrite to form a diazonium intermediate, which couples with N-(1-naphthyl)ethylenediamine to produce an azo dye. Absorbance was measured at 540 nm with a microplate reader, and the optical density (OD540), which is related to nitrite content, was recorded for each sample and reported directly. All animals received the same bronchoalveolar lavage procedure and volume. So, it is not further normalized to BALF volume and protein concentration. The absorbance at 540 nm was quantified with the Synergy HTX multimode microplate reader from BioTek, Winooski, VT, USA [27].

### 2.8. Assessment of Pulmonary Edema

Pulmonary edema was assessed by determining the lung wet-to-dry-weight ratio [28] Immediately after euthanasia, the left lung lobe was dissected, and its wet weight was recorded. The lobe was then dried in an oven at 56 °C for 48 h until a constant weight was obtained. The wet/dry weight ratio was calculated to quantify lung water content [29].

### 2.9. OVA-Specific IgE Determination

OVA-specific IgE refers to immunoglobulin E antibodies produced in response to OVA, a key marker of Th2-mediated allergic response. Serum OVA-specific IgE levels were measured using a commercial enzyme-linked immunosorbent assay (ELISA) kit (Rat Ovalbumin Specific IgE ELISA Kit, MyBiosource^®^, San Diego, CA, USA Catalog #MBS702479) according to the manufacturer’s instructions. Absorbance was read at 450 nm using a microplate reader (BioTek^®^ Synergy HTX).

### 2.10. RNA Extraction and Quantitative Real-Time PCR (qPCR)

Total RNA was isolated from homogenized lung tissue using TRIzol^®^ reagent (Invitrogen^®^, Thermo Fisher Scientific, Inc., USA). The concentration and purity of extracted RNA were measured using a NanoDrop^®^ spectrophotometer (Thermo Scientific, USA). Subsequently, 1 ug of total RNA was reverse transcribed into complementary DNA (cDNA) using the RevertAid First Strand cDNA Synthesis Kit (Thermo Scientific, USA). Gene expression analysis was conducted using the quantitative real-time PCR with SYBR^®^ Green Master Mix (Thermo-Scientific, USA) using a real-time PCR detection system. The reaction conditions were as follows: initial denaturation at 95 °C for 5 min, followed by 40 cycles of denaturation at 95 °C for 5 s, annealing at gene-specific temperature for 15 s, and extension at 72 °C for 15 s. Relative gene expression of target genes was normalized to GAPDH and calculated using the 2^−ΔΔCq^ method and expressed as log2 fold change. For gene expression data, statistical comparisons among groups were conducted using ΔCt values in keeping with conventional qPCR analysis methodology. Primer specificity was confirmed by the melt curve analysis. The corresponding nucleotide sequences for the rat-specific primers are enumerated in Table 1.

### 2.11. Lung Tissue Histopathology

For histopathological examination, the right lung lobe was inflated and fixed in 10% neutral-buffered formalin for 48 h. The tissues were then subsequently dehydrated through a graded ethanol series, which is embedded in paraffin, and sectioned at a thickness of 5 µm. The obtained sections were stained with Hematoxylin and Eosin (H&E) using standard histological procedures. All slides were coded and evaluated by a single observer who was fully blinded throughout the scoring. For each rat, three randomly selected, non-overlapping fields were evaluated for peribronchial inflammatory cell infiltration (eosinophils, neutrophils, lymphocytes, macrophages) and alveolar wall thickening. A semi-quantitative scoring system (0–3) was applied to evaluate each histological parameter (with scores assigned as 0 = absent, 1 = mild, 2 = moderate, 3 = marked based on the inflammation severity) [24]. The score was then subsequently averaged across all three fields to obtain a total airway inflammation score per animal (range 0–3).

### 2.12. Statistical Analysis

All data are expressed as mean ± standard deviation (SD) for *n* = 8 rats per group. Gene expression data were analyzed per animal using one-way ANOVA with Tukey’s post hoc test using ΔCt values. Results are provided as log2 fold change (mean ± SD) for simple interpretation. Data were analyzed using GraphPad Prism (version 9.0). Differences among groups were evaluated using one-way analysis of variance followed by Tukey’s post hoc test for multiple comparison. A probability value of less than 0.05 (*p* < 0.05) was considered statistically significant; the significance thresholds are represented symbolically in the figures and tables, as described in relevant legends.

## 3. Results

### 3.1. CN Normalizes Inflammatory Cells in Blood and BALF Under Pharmacological IDO Inhibition

Allergen challenge induced pronounced systemic and pulmonary inflammation, characterized by leukocytosis and shift in differential leukocyte composition, including increased lymphocytes and eosinophils and reduced neutrophils and monocyte percentage. In contrast, 1-MT treatment increased the neutrophil percentage in both the blood and BALF, indicating a shift away from the Th2-associated pattern seen with OVA alone. Furthermore, 1-MT alone produced only partial normalization, particularly in monocyte and eosinophil populations. CN, alone or in combination with 1-MT, effectively reduced the OVA-induced leukocytosis in blood and BALF and shifted differential leukocyte distribution, including lymphocytes, monocytes, and eosinophils towards baseline (Table 2). There was no significant difference observed in BALF neutrophil percentage between CN and CN+1-MT, but both were significantly lower than 1-MT monotherapy (CN vs. 1-MT, *p* < 0.001; 1-MT vs. CN+1-MT, *p* < 0.001), indicating that CN’s suppression of airway neutrophil recruitment is retained even after pharmacological IDO inhibition. In blood, CN+1-MT did not differ significantly from 1-MT for neutrophil count.

### 3.2. CN Suppressed Delayed-Type Hypersensitivity When IDO Activity Was Disrupted

**Figure 2:**
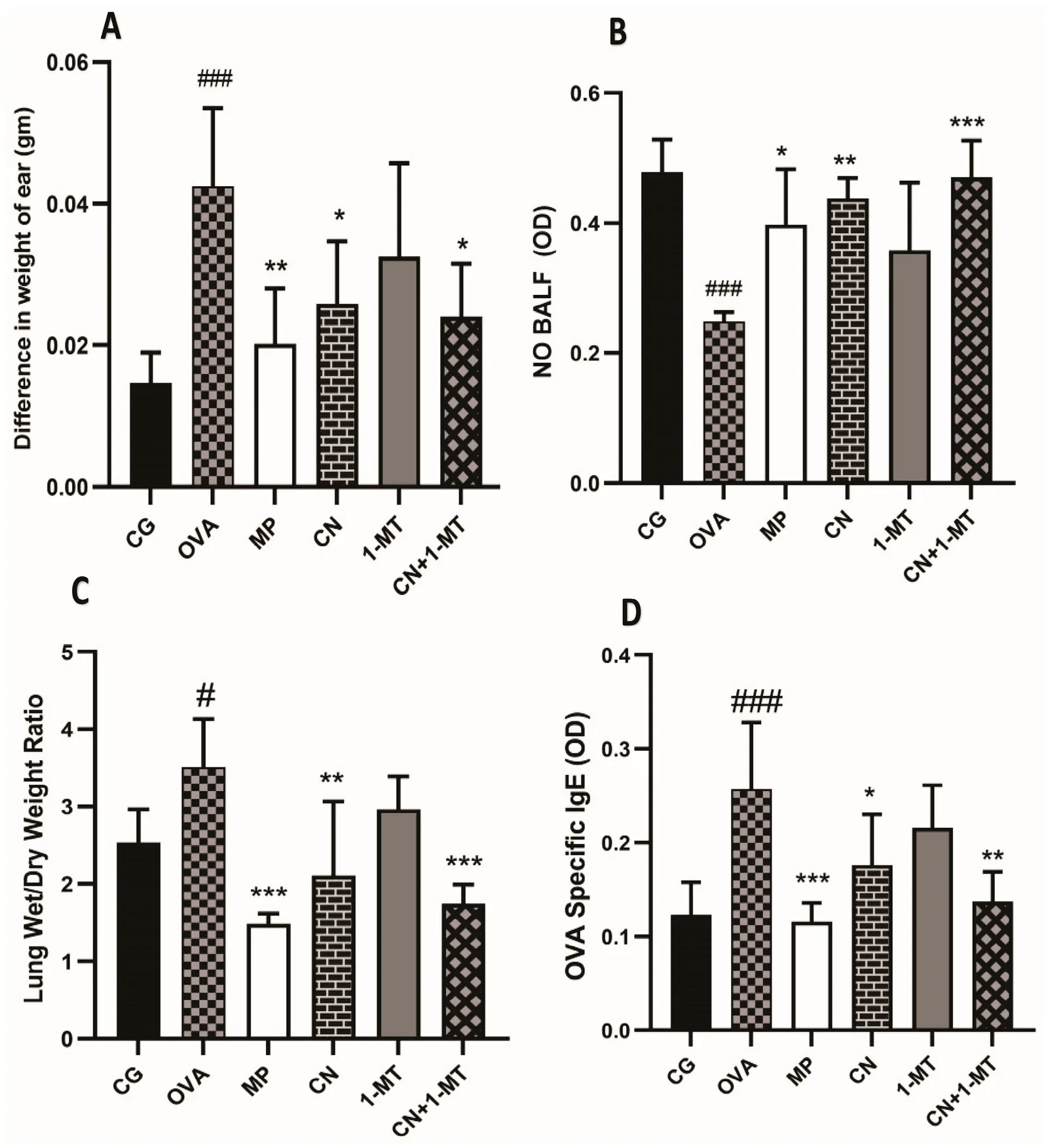
Effect of treatments on inflammatory response and immune markers. (**A**) Delayed-type hypersensitivity (DTH) response, calculated as weight difference between OVA- and PBS-injected ears. (**B**) Nitric oxide (NO) BALF levels (optical density, OD). (**C**) Lung wet-to-dry-weight ratio. (**D**) Ovalbumin (OVA)-specific IgE (OD). Data are reported as mean ± SD (*n* = 8 per group). Significance was analyzed by one-way ANOVA with post hoc test. (#, *p* < 0.05), and (###, *p* < 0.001) show comparison vs. control group (CG); (*, *p* < 0.05), (**, *p* < 0.01), and (***, *p* < 0.001) show comparison vs. Ovalbumin group (OVA). BALF, MP, CN and 1-MT represent bronchoalveolar lavage fluid, methylprednisolone, Curcumin and 1-methyl-DL-tryptophan (IDO inhibitor), respectively.

DTH is a T-cell-mediated response to an antigen. OVA challenge markedly increased the DTH response (0.042 ± 0.011 g) compared with CG (0.015 ± 0.004 g). Treatment with CN+1-MT significantly reduced this response (0.024 ± 0.008 g), comparable to MP (0.020 ± 0.008 g) and CN (0.026 ± 0.009 g) relative to OVA (Figure 2A). These data demonstrate that CN attenuates the DTH response even when IDO activity is pharmacologically inhibited; further claims of T-cell regulatory effectiveness would need direct evaluation of T-cell numbers, proliferation, or regulatory T-cell activity.

### 3.3. CN Restored Airway Nitric Oxide Production Under Pharmacological IDO Inhibition

OVA challenge significantly decreased BALF NO levels (0.249 ± 0.014 OD) compared with controls (0.479 ± 0.049 OD). Treatment with MP (0.397 ± 0.085 OD), CN (0.439 ± 0.031 OD) and CN + 1-MT (0.471 ± 0.058 OD) restored NO levels, whereas treatment with 1-MT alone showed a value of 0.358 ± 0.104 OD, which did not differ significantly from the OVA group (Figure 2B). This suggests that CN preserves airway redox balance under pharmacological IDO inhibition.

### 3.4. CN Reduced Pulmonary Edema When IDO Was Pharmacologically Suppressed

OVA-challenged rats exhibited significant pulmonary edema, with an increased wet/dry weight ratio (3.51 ± 0.62 vs. CG: 2.54 ± 0.43). Treatment with CN+1-MT significantly reduced edema (1.74 ± 0.25), comparable to MP (1.48 ± 0.14) and CN (2.11 ± 0.96) in comparison with OVA (Figure 2C). This suggests that CN reduces pulmonary edema when IDO is pharmacologically inhibited. Because the wet/dry weight ratio reflects lung water content rather than a direct measure of vascular permeability, this finding is best interpreted as decreased fluid accumulation rather than confirmed retention of endothelial barrier integrity.

### 3.5. CN Decreased the Boosted OVA-Specific IgE Levels in the Serum

The OVA group exhibited a significantly elevated level of OVA-specific IgE (0.26 ± 0.071 OD) in comparison with the CG (0.12 ± 0.035 OD), consistent with an allergen-specific humoral response. Treatment with CN+1-MT suppressed IgE (0.14 ± 0.032 OD) comparable to MP (0.12 ± 0.020 OD) and CN (0.18 ± 0.054 OD) relative to OVA (Figure 2D). These results show that CN decreases OVA-specific IgE production even when IDO is inhibited pharmacologically, indicating that CN modulates the humoral allergic response in the absence of direct evidence for B-cell activation, differentiation, or IgE class switching mechanisms.

### 3.6. CN Suppresses Cytokine Gene Expression Under Pharmacological IDO Inhibition

**Figure 3:**
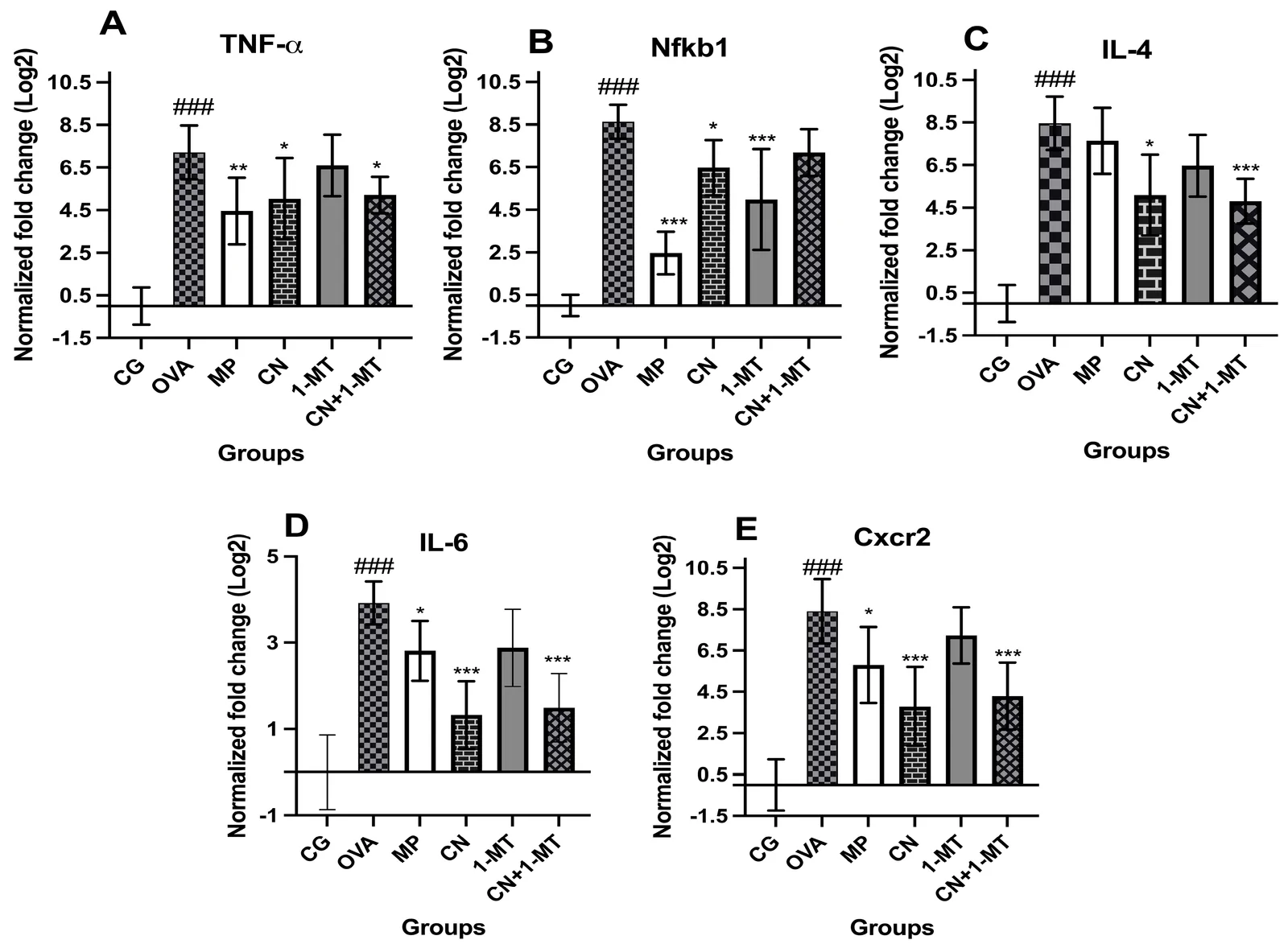
The mRNA expression levels of pro-inflammatory mediators were quantified by reverse-transcription quantitative polymerase chain reaction (RT-qPCR), with glyceraldehyde-3-phosphate dehydrogenase (GAPDH) used as reference gene. Effect of the treatments on (**A**) tumor necrosis factor-alpha (TNF-α). (**B**) NF-κB subunit 1 (Nfkb1) (**C**) Interleukin-4 (IL-4). (**D**) IL-6 and (**E**) *Cxcr2*. The data are reported as mean ± SD (n = 8 per group). Significance was analyzed by one-way ANOVA with post hoc test. (###, *p* < 0.001) show comparison vs. control group (CG); (*, *p* < 0.05), (**, *p* < 0.01), and (***, *p* < 0.001) show comparison vs. Ovalbumin group (OVA). BALF, MP, CN and 1-MT represent bronchoalveolar lavage fluid, methylprednisolone, Curcumin and 1-methyl-DL-tryptophan (IDO inhibitor), respectively.

OVA challenge induced significant transcriptional upregulation of inflammation-related genes, as compared to CG (*p* < 0.001 for all), including TNF-α, which increased to a log2 fold change (7.21 ± 1.26, Figure 3A), Nfκb1 (8.63 ± 0.80, Figure 3B), IL-4 (8.46 ± 1.25, Figure 3C), IL-6 (3.92 ± 0.50, Figure 3D), and *Cxcr2* (8.40 ± 1.56 Figure 3E), consistent with the Th2-dominant inflammatory profile.

Therapeutic interventions markedly attenuated these responses with differing efficacy patterns. MP significantly reduced the expression of TNF-α (4.46 ± 1.57, Figure 3A; *p* < 0.01 vs. OVA), Nfkb1 (2.47 ± 1.00, Figure 3B; *p* < 0.001 vs. OVA), IL-6 (2.81 ± 0.69, *p* < 0.05 vs. OVA Figure 3D) and *Cxcr2* (5.80 ± 1.84, Figure 3E; *p* < 0.05 vs. OVA), but non-significant results were observed in IL-4 (7.64 ± 1.55, Figure 3C) relative to OVA. CN produced significant downregulation of TNF-α (5.04 ± 1.91, Figure 3A; *p* < 0.05), Nfkb1 (6.47 ± 1.30, Figure 3B; *p* < 0.05), IL-4 (5.09 ± 1.90, Figure 3C; *p* < 0.05), IL-6 (1.33 ± 0.79, Figure 3D; *p* < 0.001), and *Cxcr2* (3.79 ± 1.91, Figure 3E; *p* < 0.001). 1-MT monotherapy significantly reduced the expression of Nfkb1 (4.98 ± 2.37, Figure 3B; *p* < 0.001 vs. OVA) but produced no significant change in TNF-α (6.60 ± 1.45, Figure 3A), IL-4 (6.47 ± 1.45, Figure 3C), IL-6 (2.88 ± 0.90, Figure 3D), or *Cxcr2* (7.23 ± 1.37, Figure 3E) relative to OVA. Notably, CN+1-MT elicited a significant reduction in the expression of TNF-α (5.21 ± 0.86, Figure 3A; *p* < 0.05), IL-4 (4.81 ± 1.05, Figure 3C; *p* < 0.001), IL-6 (1.50 ± 0.79, Figure 3D; *p* < 0.001), and *Cxcr2* (4.29 ± 1.62, Figure 3E; *p* < 0.001) relative to OVA, whereas the reduction in Nfkb1 expression did not reach statistical significance (7.19 ± 1.10, Figure 3B). Both treatments considerably exceeded 1-MT monotherapy for IL-4, IL-6, and *Cxcr2* (Figure 3C–E), but pairwise comparisons between CN and CN+1-MT revealed no significant difference at any of the five targets (Figure 3A–E). These transcriptional alterations show decreased expression of genes linked to inflammation, but they do not by themselves show inhibition of NF-κB pathway activity. Instead, protein-level or functional evaluation is needed.

### 3.7. Histopathological Protection Sustained by CN Under IDO-Compromised Condition

**Figure 4:**
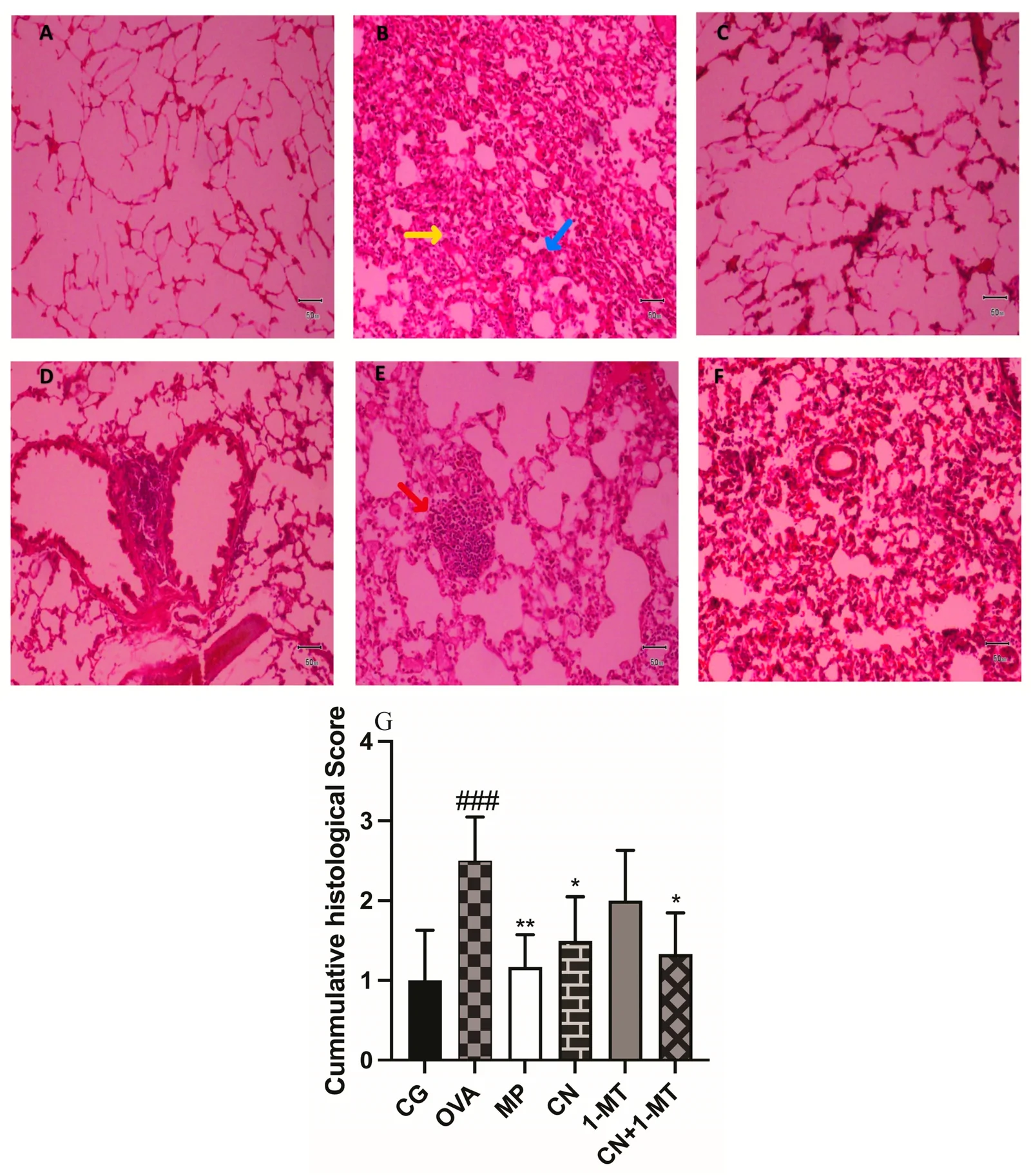
Histopathological assessment of lung tissues. Representative Hematoxylin and Eosin (H&E)-stained lung section (10X magnification). Scale Bar: 50 μm. (**A**) Control group (CG) showing normal alveolar architecture. (**B**) Ovalbumin (OVA) challenge shows lungs with peribronchial and perivascular inflammatory infiltrates (yellow arrow), epithelial desquamation, and marked alveolar wall thickening (blue arrow). (**C**) Methylprednisolone (MP) and (**D**) Curcumin (CN) markedly reduced inflammatory infiltrates and preserved alveolar morphology. (**E**) 1-methyl-DL-tryptophan (1-MT) showing neutrophil-predominant infiltration (red arrow) and alveolar wall thickening. (**F**) Curcumin plus 1-methyl-DL-tryptophan (CN+1-MT) markedly reduced inflammatory infiltrates and preserved alveolar morphology. (**G**) Quantitative histological scoring of inflammation. Data are reported as mean ± SD (*n* = 8 per group). Statistical significance. (###, *p* < 0.001) shows comparison vs. control group (CG); (*, *p* < 0.05) and (**, *p* < 0.01) show comparison vs. Ovalbumin group (OVA). BALF, MP, CN and 1-MT represent bronchoalveolar lavage fluid, methylprednisolone, Curcumin and 1-methyl-DL-tryptophan (IDO inhibitor), respectively.

Histological examination showed normal alveolar architecture in CG, with thin septa and minimal inflammatory infiltrates. The OVA-challenged group displayed dense peribronchial and perivascular infiltration of eosinophils, neutrophils, lymphocytes, and macrophages, accompanied by epithelial desquamation, distortion of alveolar structure and marked alveolar thickening (blue arrow). MP markedly reduced inflammatory cell infiltration and preserved alveolar morphology. CN produced similar protection, with substantially fewer inflammatory cells and preserved alveolar integrity. In contrast, 1-MT treatment led to neutrophil-predominant inflammatory foci (red arrow), persistent interstitial inflammation, and pronounced alveolar wall thickening, consistent with a neutrophil-predominant inflammatory pattern associated with IDO suppression. Notably, CN+1-MT attenuated these changes, showing reduced inflammatory cell infiltration and less thickening in alveolar walls (Figure 4A–E). Composite histological scores were significantly elevated in the OVA group (2.50 ± 0.547; *p* < 0.001) but reduced by MP (1.17 ± 0.41) and CN (1.50 ± 0.55). The 1-MT group showed persistent neutrophil-dominant inflammation (2.00 ± 0.63), whereas CN and CN+1-MT treatments (1.50 ± 0.55 and 1.33 ± 0.52, respectively) significantly mitigated inflammation (Figure 4G). These findings indicate that CN effectively mitigates airway injury and restrains neutrophil-dominant inflammation even under pharmacological IDO inhibition, consistent with reduced inflammatory cell infiltration and tissue injury under compromised immunoregulation.

## 4. Discussion

This study found that CN preserved anti-inflammatory and tissue-protective properties in an OVA-induced model of allergic airway inflammation when IDO was pharmacologically inhibited. The results show that CN remained effective under these experimental settings; however, the molecular processes behind this observation were not explicitly examined. CN, alone or in combination with 1-MT, effectively mitigated systemic and pulmonary leukocytosis, normalized differential leukocyte counts, suppressed adaptive immune responses, attenuated vascular leak, downregulated pro-inflammatory cytokines, and improved histopathological scores. These findings extend existing knowledge by showing that CN’s anti-inflammatory potential remains functionally preserved under conditions of compromised IDO-mediated immune tolerance, which may mirror immune-metabolic dysregulation in corticosteroid-resistant or neutrophilic asthma. Although CN’s inhibition of NF-κB/MAPK signaling is well-established [30,31], this study adds a novel functional perspective by demonstrating that CN’s anti-inflammatory effectiveness is maintained when IDO-mediated tryptophan catabolism is pharmacologically blocked. Additional mechanistic research is needed to identify whether these effects include different immunometabolic pathways.

Interestingly, the MP and CN+1-MT groups had wet/dry weight ratios lower than the CG baseline. Methylprednisolone and other glucocorticoids activate ENaC expression and Na^+^/K^+^-ATPase activity in alveolar epithelium cells, leading to improved alveolar fluid clearance rather than just preventing edema formation. Pretreatment with dexamethasone has been shown to increase alveolar fluid clearance by up to 80% compared to untreated lungs [32]. This process most likely explains the sub-baseline ratio reported with MP. CN+1-MT exhibited a similar trend, which might indicate a similar, though not as mechanistically characterized, effect of CN on pulmonary fluid management. In order to corroborate this mechanism, further research would require evaluation of ENaC expression or alveolar fluid clearance.

Hematological analysis confirmed that OVA sensitization induced classical Th2-type inflammation characterized by leukocytosis, eosinophilia, lymphocytosis and neutropenia, whereas IDO inhibition shifted the inflammatory profile towards neutrophil predominance [6,33]. Interestingly, in the CN, 1-MT, and CN+1-MT groups, total blood leukocyte counts were lower than the CG value. The CN+1-MT group had the lowest count, whereas 1-MT monotherapy was middle (Table 2). This pattern cannot be attributable to CN’s direct pharmacological impact, because the 1-MT monotherapy group received no CN and went below baseline. The fact that the combination group had the lowest numerical score is consistent with the two treatments working in combination; however, the current one-way design was not meant to assess an interaction, and no such claim is being made. The differential profile suggests redistribution rather than suppressed leukopoiesis; the decline was driven primarily by lymphocytes and monocytes, while neutrophil fractions increased in blood and BALF, with neutrophil-predominant foci visible histologically, consistent with leukocyte differentiation and mobilization into inflamed pulmonary tissue rather than reduced marrow output [34]. IDO blockage provides a reasonable, CN-independent contribution. Kynurenine-pathway metabolites induce T-cell death and inhibit lymphocyte proliferation [35,36], and downstream *AhR* signaling modulates hematopoietic progenitor activity [37]; inhibiting these pathways under 1-MT may reduce circulating lymphocyte numbers independent of CN. Repeated daily intraperitoneal injection is another non-specific component, since handling stress causes glucocorticoid-mediated leukocyte redistribution [38], which is common to all treatment groups but lacking in CG. Because kynurenine, tryptophan, and bone marrow production were not tested, these pathways may only be considered as hypotheses. Plasma kynurenine to tryptophan ratios, and direct marrow measurement, are required to separate redistribution from inhibited production to balance IDO blocking against procedural stress.

In order to verify Th1 or Th17 polarization, direct markers, like IFN-γ, IL-17, or T-bet, were not evaluated in this investigation. CN treatment suppressed eosinophilic infiltration and rebalancing lymphocytes under these conditions. The pharmacology of 1-MT itself must temper this interpretation: it is a competitive, not irreversible, inhibitor of IDO, and even at saturating concentrations, functional reversal of IDO-mediated suppression by 1-MT is usually incomplete because IDO has a greater affinity for tryptophan than 1-MT does. Additionally, 1-MT has been shown to have IDO-independent effects, such as modulating TLR/MAPK signaling in myeloid cells and directly activating the aryl hydrocarbon receptor (AhR) [39,40,41,42]. This study, therefore, describes CN’s effect as retained under pharmacological IDO challenge rather than as an IDO-independent mechanism because our data cannot differentiate a truly IDO independent CN effect from residual IDO-pathway activity or from 1-MT off-target effects due to this incomplete and non-specific inhibition. One possible compensating pathway is CN’s direct *AhR* interaction, which is independent of kynurenine production. CN has been shown to activate *AhR* signaling in inflammatory conditions, through the formation of the Nrf2-Jdp2-*AhR* transcriptional complex [43], and *AhR* activation is sufficient to inhibit Th17/IL-17A-driven, neutrophil-associated inflammation in lung injury models [44]. This suggests a testable hypothesis: CN may partially bypass the loss of IDO/kynurenine signals by directly activating *AhR* rather than tryptophan catabolites. The current study does not examine this, but it provides a clear route for future research (by measuring *AhR* reporter assays, CYP1A1/CYP1B1 induction, and tryptophan/kynurenine ratio).

Allergic airway inflammation critically involves eosinophils. These cells contribute by secreting cytotoxic substances, including major basic protein and eosinophil peroxidase, along with cytokines like IL-5 and eotaxin that enhance Th2 immune responses [45,46]. CN markedly reduced eosinophil infiltration, consistent with its previously described inhibition of IL-5 and eotaxin expression [21]. Conversely, IDO inhibition promoted neutrophil accumulation, which is consistent with neutrophilic-predominant inflammation. Th1/Th17 skewing has been reported under IDO blockade [8,47]. The ability of CN+1-MT to normalize leukocyte composition suggests a mechanistic engagement not solely dependent on IDO, enabling CN to modulate leukocyte composition despite pharmacological inhibition of IDO. However, the absence of data on kynurenine metabolites, aryl hydrocarbon receptor signaling, or Th17-markers limits mechanistic interpretation and represents a key direction for future research to validate the specific pathways underlying this effect.

OVA challenge reduced BALF NO levels, whereas CN restored NO levels even under an IDO-compromised condition. BALF nitrite indicates a different NO pool, despite the fact that exhaled NO (FeNO) is often high in allergic asthma. Allergen challenge increases arginase activity, which depletes the shared substrate L-arginine, uncoupling NOS isoforms, including iNOS, and generating superoxide and peroxynitrite instead of NO, resulting in a net reduction in airway NO output regardless of persistent inflammation [48,49]. This arginase-mediated NO shortage results in decreased airway smooth muscle relaxation and hyperresponsiveness, and restoring NOS-derived NO with eNOS overexpression reduces airway inflammation and hyperresponsiveness in a mouse asthma model [50]. The current data support modification of the arginase-NOS axis, although direct measurements of arginase activity, NOS isoform expression, or FeNO would be required to validate this.

Histological findings mirrored systemic observations. OVA-challenged rats displayed peribronchial and perivascular infiltration of eosinophils, neutrophils, lymphocytes, macrophages, epithelial desquamation, and alveolar wall thickening, whereas 1-MT administration sustained neutrophilic inflammation and tissue injury consistent with IDO suppression [8]. CN alone or combined with 1-MT markedly reduced inflammatory cell recruitment and preserved tissue architecture, demonstrating its protective effect against epithelial injury and potential to prevent airway remodeling. By limiting neutrophil accumulation and oxidative injury, CN may disrupt the inflammation and remodeling continuum driven by proteolytic enzymes (e.g., elastase, MMPs), reactive oxygen species and profibrotic mediators such as TGF-β [51,52].

Adaptive immune responses were also modulated by CN. DTH and OVA-specific IgE were significantly reduced by CN+1-MT, demonstrating suppression of cellular and humoral arms of the allergic response under pharmacological IDO inhibition. CN has been shown to decrease T-cell proliferation, IL-2 and IFN-γ production, and B-cell differentiation via NF-κB-dependent pathways in various experimental systems [53,54]. Such mechanisms were not directly investigated in the current study but are described here as potential pathways underlying the reported immunomodulatory effects.

At the molecular level, treatment with CN and CN +1-MT resulted in significant suppression of TNF-α, IL-4, IL-6 and *Cxcr2*. CN alone significantly reduced the expression of pro-inflammatory mediator Nfkb1 (Figure 3A–E). The improvement observed in the histological and immunological measures is consistent with these molecular changes, but they do not by themselves demonstrate a mechanistic foundation. CN is well documented to inhibit the NF-κB signaling cascade by preventing IκBα phosphorylation and nuclear translocation of the p65 subunit, thereby attenuating transcription of downstream inflammatory cytokines [19,55].

Because rats do not have a canonical IL-8/CXCL8 ortholog, the functional neutrophil-chemoattractant signal in rats is mediated by CXCL1 and CXCL2 that acts on their shared receptor, *Cxcr2*, which is expressed on neutrophils and airway epithelial cells and also drives neutrophil chemotaxis into inflamed tissue [56,57]. This study found that OVA exposure increased the *Cxcr2* transcript relative to CG (Figure 3E), and CN treatment reduced this, which corresponded to the decrease in BALF and blood neutrophil infiltration seen in Table 2. This is consistent with previous research demonstrating that CN significantly reduced levels of keratinocyte-derived chemokine (KC/CXCL1, a primary *Cxcr2* ligand) in bronchoalveolar lavage fluid, with a corresponding reduction in neutrophil recruitment in airway inflammation mouse models. In comparison to untreated controls, CN decreased neutrophils by 87% and BALF KC by 80% [57]. Additionally, CN’s wider inhibitory effects on chemokine and chemokine-receptor signaling throughout inflammatory situations have been summarized in a recent study [58]. CN suppresses *Cxcr2*-axis signaling by inhibiting NF-κB-dependent transcription of its upstream ligands (CXCL1/CXCL2). This impact requires intact IκBα activity [59]. Taken together, the reduction in *Cxcr2* expression by CN, alone or in combination with 1-MT, is consistent with reduced neutrophil chemotactic signaling as a contributor to the reduced neutrophilic infiltration observed in BALF and lung histology, though direct measurement of CXCL1/CXCL2 protein, receptor occupancy, or *Cxcr2*-dependent chemotaxis was not performed and would be required to confirm this pathway directly. The statistical differentiation between CN/CN+1-MT and 1-MT was only seen in BALF neutrophils, not in blood. Airway neutrophilic inflammation is controlled centrally by chemokine-gradient-mediated recruitment into the airway lumen via the CXCL1/CXCL2, *Cxcr2* axis, as previously stated, whereas peripheral blood neutrophil numbers are controlled by systemic granulopoiesis and marginal pool movements. CN’s impact is most statistically significant in the airway compartment, where *Cxcr2*-mediated inflammatory recruitment occurs, which supports a local, chemokine-driven mode of action on airway neutrophilic inflammation under pharmaceutical IDO inhibition. This compartment-specific pattern reflects findings from blood and airway-level leukocyte measurements deviating from each other in human asthma [60].

*Cxcr2* has been specifically targeted in clinical studies of neutrophilic asthma and COPD, making it more than just a preclinical marker of neutrophilic airway diseases. According to Nair et al. (2012), *Cxcr2* is a therapeutically actionable regulator of airway neutrophil recruitment in human diseases since a *Cxcr2* antagonist dramatically decreased sputum neutrophil counts in individuals with severe asthma and sputum neutrophilia [61]. Even while *Cxcr2*-driven neutrophil recruitment is mechanistically significant, it is insufficient to prevent clinical exacerbations on its own, according to later more extensive studies with *Cxcr2* antagonists (such as AZD5069) that failed to lower exacerbation rates in uncontrolled chronic asthma [62]. In this regard, the decrease in *Cxcr2* transcript expression seen with CN in the current model highlights that transcriptional suppression of *Cxcr2* alone should not be assumed to translate into a proportionate clinical benefit, while additionally demonstrating a particular, clinically validated axis through which CN may influence neutrophil-associated airway pathology.

Similarly, IL-6 overexpression exhibited its pivotal role in amplifying Th2/Th17 differentiation, IgE class switching, and airway hyperresponsiveness [63,64], and it was significantly attenuated by CN and CN+1-MT. Although NF-κB/*STAT3* signaling was not explicitly evaluated in this study, previous results [65,66] suggest that this route may have contributed to the CN-driven IL-6 suppression seen here, and further investigation is necessary. The low impact of 1-MT alone on IL-6 is consistent with CN’s anti-inflammatory action, persisting even after pharmacological IDO suppression [67,68]. Among the five targets assessed, Nfkb1 did not reach significance in the CN+1-MT group, but this should not be interpreted as loss of CN efficacy. Nfkb1 encodes the p105/p50 precursor, whereas canonical NF-κB activity is primarily regulated post-translationally through IKK-dependent IκBα degradation and p65 nuclear translocation [69]. Nfkb1’s transcript level may not accurately reflect pathway activity due to its NF-κB-responsive nature and autoregulation. CN’s suggested impact on IKK inhibition and IκBα stability [70] cannot be determined only from Nfkb1 expression. Consistent with this, 1-MT lowered Nfkb1 without affecting downstream inflammatory mediators. Moreover, p50 homodimers are lacking a transactivation domain and operate as transcriptional repressors at κB sites, in part through recruitment of HDAC-1 [71,72]. Therefore, a reduction in the Nfkb1 transcript does not simply transfer into lower inflammatory output. At the existing sample size, type II errors cannot be eliminated. The data suggest suppressing downstream inflammation but do not directly block the NF-κB pathway. This requires a measurement of IκBα phosphorylation, nuclear p65, and NF-κB transcriptional activity.

These results align with previous studies showing CN’s effectiveness in OVA-induced airway inflammation. In a study of OVA-sensitized mice, Oh et al. found that administering CN at the same dose (200 mg/kg, i.p.) reduced total leukocyte and eosinophil counts in BAL fluid, decreased BALF IgE, and attenuated airway hyperresponsiveness. They attributed these effects to NF-κB inhibition with IκBα stabilization and blockade of p65/p50 nuclear translocation. In the current study, the same direction of impact is shown in the decreased TNF-α, IL-4, and Nfkb1 transcript expression, IgE suppression, and eosinophilic infiltration. In a chronic OVA-asthma model, Zhu similarly showed that CN decreased BALF TNF-α, IL-4, IL-5, and IL-13 along with inflammatory cell infiltration via a PPARγ-dependent NF-κB pathway, which is again consistent with the cytokine suppression shown in the present research [21,73]. The current results add to the body of work by demonstrating that CN’s effectiveness across leukocyte, cytokine, and histological endpoints is maintained even when IDO-mediated immune tolerance is pharmacologically disrupted with 1-MT, in contrast to studies that looked at CN monotherapy in immunologically normal animals.

The current study has a few limitations. IDO inhibition by 1-MT was determined pharmacologically rather than biochemically; kynurenine, tryptophan, their ratio, and enzymatic activity were not examined. Furthermore, 1-MT is a partial, competitive IDO inhibitor with documented IDO-independent effects such as *AhR* activation and TLR/MAPK signaling modification [41,42]. As a result, the 1-MT condition is a mixed pharmacological state, and data attributable only to IDO blockage should be evaluated carefully. The immunological examination was inadequate, since IL-5, eotaxin, IFN-γ, IL-17, and airway hyperresponsiveness were not tested. Differential leucocyte counts are compositional and, hence, not statistically independent; changes in one population always impact the proportionate representation of others and, thus, should be analyzed with total leukocyte counts (Table 2). CN was given in 5% DMSO without a DMSO-only control, which prevented full exclusion of vehicle-related effects. Ether was utilized for terminal anesthetic consistently across groups under the approved protocol, albeit isoflurane would be preferred in future research, including lavage and airway histology. The histological grading was performed by a single blinded observer; therefore, inter-operator reliability could not be determined. Additionally, the downstream NF-κB/MAPK signaling intermediates were not directly analyzed. Thus, the mechanistic results remain hypothesis generating and should be validated utilizing genetic techniques, metabolomic profiling, protein-level signaling tests, and blinded multi-observer histological scoring with verified concordance.

## 5. Conclusions

In conclusion, CN decreased various inflammatory, immunological, and histological parameters of OVA-induced airway inflammation when given alone or in combination with pharmaceutical-based IDO inhibition. Although the present research shows that CN is effective under these experimental circumstances, it was not intended to elucidate the particular chemical pathways involved. Future investigations should include direct assessments of IDO activity, kynurenine metabolism, and downstream signaling pathways.

## Figures and Tables

**Table 1:** Sequences and optimized annealing temperature of primers used in qRT-PCR assays.

Primers	Forward/Reverse	Sequence (5′-3′)	Cycles	Annealing Temp. (°C)
TNF-α	Forward Reverse	AAGCTGTCTTCAGGCCAACA CCCGTAGGGCGATTACAGTC	40	59
NF-κB	Forward Reverse	CAAGGAAGAGGATGTGGGGTT AGCTGAGCATGAAGGTGGATG	40	60
IL-4	Forward Reverse	AACACCACGGAGAACGAGCTCATC AGTGAGTTCAGACCGCTGACACCT	40	58
*Cxcr2*	Forward Reverse	CCACGCCACAAGTACACTGAT TGGTTCTCATGAGGGTGTCTG	40	57
IL-6	Forward Reverse	CCCACCAAGAACGATAGTCA CTCCGACTTGTGAAGTGGTA	40	58
GAPDH	Forward Reverse	TCTCTGCTCCTCCCTGTTCT CTTGCCGTGGGTAGAGTCAT	40	60

Quantitative real-time PCR (qPCR) was performed using specific forward and reverse primers for the target genes TNF-α, Nfkb1, IL-4, IL-6, and *Cxcr2*, with GAPDH serving as the reference gene. Each reaction was performed over 40 amplification cycles, with the annealing temperatures optimized for primer specificity and amplification efficiency.

**Table 2:** Effect on total and differential leukocyte counts in blood and bronchoalveolar lavage fluid following therapeutic intervention.

Treatment	Total Cell Count in Blood × 10^3^/µL	Differential Cell Count in Blood (%)			
		**Lymphocytes**	**Neutrophils**	**Monocytes**	**Eosinophils**
**CG**	5.33 ± 0.48	60.42 ± 3.61	28.91 ± 4.56	7.32 ± 1.38	0.90 ± 0.25
**OVA**	8.90 ± 2.19 ###	78.44 ± 6.11 ###	15.68 ± 6.03 ##	2.11 ± 1.96 ###	3.68 ± 0.97 ###
**MP**	5.03 ± 0.66 ***	63.78 ± 4.38 ***	28.62 ± 5.78 *	4.98 ± 0.45 *	0.79 ± 0.52 ***
**CN**	3.55 ± 1.45 ***	68.02 ± 3.52 *	23.52 ± 3.078	5.42 ± 1.38 **	1.46 ± 0.09 ***
**1-MT**	2.54 ± 0.40 ***	59.45 ± 8.99 ***	36.43 ± 10.15 ***	1.73 ± 2.16	1.42 ± 0.74 ***
**CN+1-MT**	1.64 ± 0.96 ***	66.75 ± 3.75 **	26.29 ± 4.13 *	2.55 ± 0.96	2.17 ± 0.71 **
**Treatment**	**Total Cell Count in BALF × 10^3^/µL**	**Differential cell count in BALF (%)**			
		**Lymphocytes**	**Neutrophils**	**Monocytes**	**Eosinophils**
**CG**	0.41 ± 0.10	49.93 ± 5.22	41.73 ± 5.11	3.93 ± 2.51	3.45 ± 1.80
**OVA**	5.96 ± 1.55 ###	36.46 ± 5.35 #	25.03 ± 2.42 ##	26.95 ± 6.54 ###	10.85 ± 3.17 ###
**MP**	0.27 ± 0.22 ***	54.53 ± 5.89 ***	31.11 ± 3.57	10.67 ± 2.81 ***	3.01 ± 0.58 ***
**CN**	0.41± 0.23 ***	51.49 ± 6.65 **	28.61 ± 8.12	16.71 ± 6.03 *	2.38 ± 0.95 ***
**1-MT**	0.30 ± 0.11 ***	31.32 ± 5.38	54.90 ± 5.64 ***	7.55 ± 3.27 ***	5.89 ± 1.85 ***
**CN+1-MT**	0.56 ± 0.21 ***	56.30 ± 8.61 ***	32.05 ± 10.63	9.18 ± 2.85 ***	1.76 ± 0.93 ***

Data are reported as mean ± SD, for (*n* = 8 per group). Significance was analyzed by one-way ANOVA with post-hoc test. (#, *p* < 0.05), (##, *p* < 0.01), and (###, *p* < 0.001) shows comparison vs. control group (CG); (*, *p* < 0.05), (**, *p* < 0.01), and (***, *p* < 0.001) shows comparison vs. Ovalbumin group (OVA). BALF, MP, CN and 1-MT represent bronchoalveolar lavage fluid, methylprednisolone, Curcumin and 1-methyl-DL-tryptophan (IDO inhibitor), respectively.

## Data Availability

The original contributions presented in this study are included in the article. Further inquiries can be directed to the corresponding authors.

## Abbreviations

AhR, aryl hydrocarbon receptor; ANOVA, analysis of variance; AQP, aquaporin; BALF, bronchoalveolar lavage fluid; cDNA, complementary deoxyribonucleic acid; CG, control group; CN, Curcumin; COPD, chronic obstructive pulmonary disease; *Cxcr2*, C-X-C motif chemokine receptor 2; CXCL1, C-X-C motif chemokine ligand 1; CXCL2, C-X-C motif chemokine ligand 2; DMSO, dimethyl sulfoxide; DTH, delayed-type hypersensitivity; ELISA, enzyme-linked immunosorbent assay; GAPDH, glyceraldehyde-3-phosphate dehydrogenase; ENaC, epithelial sodium channel; H&E, Hematoxylin and Eosin; ICS, inhaled corticosteroids; IDO, Indoleamine 2,3-dioxygenase; IFN-γ, interferon gamma; IgE, immunoglobulin E; IKK, IκB kinase; IκBα, inhibitor of κB alpha; IL, interleukin; iNOS, inducible nitric oxide synthase; KC, keratinocyte-derived chemokine; LABA, long-acting beta2-agonist; MAPK, mitogen-activated protein kinase; MMP, matrix metalloproteinase; MP, methylprednisolone; NF-κB, nuclear factor kappa B; NO, nitric oxide; Nrf2, nuclear factor erythroid 2-related factor 2; OVA, Ovalbumin; PAS, periodic acid–Schiff; PBS, phosphate-buffered saline; PCR, polymerase chain reaction; PPARγ, peroxisome proliferator-activated receptor gamma; qPCR, quantitative polymerase chain reaction; RNA, ribonucleic acid; RT-qPCR, real-time quantitative polymerase chain reaction; SD, standard deviation; STAT3, signal transducer and activator of transcription 3; TGF-β, transforming growth factor beta; Th1, T-helper 1; Th2, T-helper 2; Th17, T-helper 17; TLR, Toll-like receptor; TNF-α, tumor necrosis factor alpha; 1-MT, 1-methyl-DL-tryptophan.
